# Procalcitonin and procalcitonin kinetics for diagnosis and prognosis of intravascular catheter-related bloodstream infections in selected critically ill patients: a prospective observational study

**DOI:** 10.1186/1471-2334-12-247

**Published:** 2012-10-08

**Authors:** Vasiliki P Theodorou, Vasilios E Papaioannou, Gregory A Tripsianis, Maria K Panopoulou, Elias K Christophoridis, Georgios A Kouliatsis, Theodora M Gioka, Efstratios S Maltezos, Sophia I Ktenidou-Kartali, Ioannis A Pneumatikos

**Affiliations:** 1 Department of Intensive Care Unit, University Hospital of Alexandroupolis, Dragana, 68100, Alexandroupolis, Greece; 2 Department of Medical Statistics, University Hospital of Alexandroupolis, Dragana, 68100, Alexandroupolis, Greece; 3 Laboratory of Medical Microbiology, University Hospital of Alexandroupolis, Dragana, 68100, Alexandroupolis, Greece; 4 Department of Biopathology, University Hospital of Alexandroupolis, Dragana, 68100, Alexandroupolis, Greece; 5 2nd Department of Internal Medicine, University Hospital of Alexandroupolis, Dragana, 68100, Alexandroupolis, Greece

## Abstract

**Background:**

Procalcitonin (PCT) has emerged as a valuable marker of sepsis. The potential role of PCT in diagnosis and therapy monitoring of intravascular catheter-related bloodstream infections (CRBSI) in intensive care unit (ICU) is still unclear and was evaluated.

**Methods:**

Forty-six patients were included in the study, provided they were free of infection upon admission and presented the first episode of suspected CRBSI during their ICU stay. Patients who had developed any other infection were excluded. PCT was measured daily during the ICU hospitalization. Primary endpoint was proven CRBSI. Therapy monitoring as according to infection control was also evaluated.

**Results:**

Among the 46 patients, 26 were diagnosed with CRBSI. Median PCT on the day of infection suspicion (D0) was 7.70 and 0.10 ng/ml for patients with and without proven CRBSI, respectively (p < 0.001). The area under the curve (AUC) for PCT was 0.990 (95% CI; 0.972 – 1.000), whereas a cut-off value of 0.70 ng/ml provided sensitivity and specificity of 92.3 and 100% respectively. In contrast, the AUC for white blood cells (WBC) was 0.539 (95% CI; 0.369 – 0.709), and for C-reactive protein (CRP), 0.603 (95% CI; 0.438 – 0.768). PCT was the best predictor of proven infection. Moreover, an increase >0.20 ng/ml of PCT between the D0 and any of the 4 preceding days was associated with a positive predictive value exceeding 96%. PCT concentrations from the D2 to D6 after suspected infection tended to decrease in controlled patients, whereas remained stable in non-controlled subjects. A PCT concentration exceeding 1.5 ng/ml during D3 was associated with lack of responsiveness to therapy (p = 0.028).

**Conclusions:**

We suggest that PCT could be a helpful diagnostic and prognostic marker of CRBSI in critically ill patients. Both absolute values and variations should be considered.

## Background

Use of central venous catheters (CVC) is essential in caring for critically ill patients. However, despite new knowledge in the pathogenesis [1] and prevention [2,3], catheter-related bloodstream infections (CRBSIs) still remain a leading cause of health-care-associated infections [4], and are related with significant morbidity, mortality, and hospital cost [5,6].

Conventional approach to managing this type of infection requires a decision making regarding the removal or sparing of the indwelling catheter and the early institution of empirical antimicrobial therapy [6]. Furthermore, antibiogram-guided antibiotic treatment and repeated blood cultures for assessing control of infection, follows. However, the above practice is often problematic, mainly because classical criteria that are often associated with CRBSI, such as fever, chills, or hypotension are too nonspecific to establish a diagnosis [6]. In addition, the majority of CRBSI occurs in the absence of local symptoms [7]. Since critically ill patients have many reasons to develop systemic inflammatory response syndrome (SIRS), there is a tendency to assume that many patients with a CVC in place might suffer from CRBSI. Unfortunately, up to 70% of the central venous catheters removed due to suspected infection, prove to be sterile [5].

Therefore, diagnosis of CRBSI by a catheter-sparing method with a high degree of accuracy is essential. For this purpose, microbiological characteristics such as differential time to positivity, simultaneous quantitative blood cultures, or the time to positivity of blood cultures [8,9] have been suggested to be discriminative. However, time delay of several days and increased cost are considered major disadvantages. In addition, some of these techniques are labour intensive.

On the other hand, the results of therapy delay, regarding the proportion of survival, are well established in septic shock [10], and as it was suggested, prompt institution of appropriate therapy, along with removal of indwelling catheter is essential for successful infection control [11].

In this respect, such infections are often overdiagnosed, resulting in unnecessary and wasteful removal of the indwelling vascular catheter and antibiotic overuse, favoring at the same time potential emerging of multidrug resistant organisms. Unfortunately, commonly used biomarkers, such as WBC or CRP lack adequate specificity for diagnosis of bacterial infections [12].

In this context, we have hypothesized that measurement of PCT [13], which is a fast-reacting biomarker, could be a useful method for early diagnosis and therapy monitoring of CRBSIs in critically ill patients. A plethora of clinical observational studies have evaluated diagnostic accuracy of PCT for discriminating patients with SIRS versus sepsis [14,15]. In addition, several trials have shown that the use of PCT-guided algorithms towards antibiotic stewardship programs is capable to reduce the consumption of antibiotics in septic patients [16,17]. However, different meta-analyses have obtained conflicting results regarding reliability of PCT in diagnosing sepsis or bacteremia [18,19]. In this respect, it has been emphasized that PCT must always be interpreted in the context of a careful clinical and microbiological assessment [20].

There are only a few studies in the literature that have assessed the accuracy of PCT for CRBSI diagnosis [21,22]. Chen and colleagues [21] assessed PCT discriminative value in terms of CRBSI diagnosis in a group of liver transplanted patients, whereas Schuetz et al. [22] studied a cohort of medical patients with different diagnoses. However, both research groups did not mention either if they recruited patients under mechanical ventilator support, or if studies were performed in the ICU.

Thus, the aim of the present study was to evaluate the accuracy of PCT serum levels and its kinetics, before development of a suspected infection, for diagnosing CRBSI in critically ill patients during their stay in the ICU. Moreover, we tried to compare its discriminating power with other biomarkers, such as WBC count and CRP serum levels, during an episode of suspected infection. The prognostic role of serial PCT measurements as monitoring to infection control was also assessed.

## Methods

This observational cohort study was performed in a nine-bed medico-surgical ICU in the teaching hospital of Alexandroupolis, Greece, between June 2008 and May 2011. The hospital ethics committee on human research approved the study protocol and informed consent was obtained from the patients’ next of kin.

**Figure 1:**
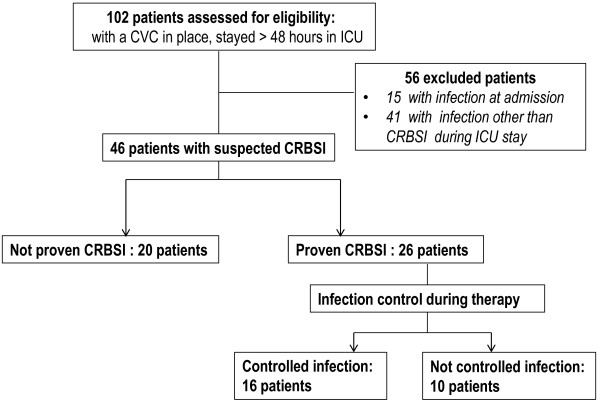
Flow chart of the study, CVC: Central venous catheter; ICU: Intensive care unit; CRBSI: Catheter - related bloodstream infection.

One hundred and 2 patients admitted to the ICU and suspected to stay longer than 48 h with a central vascular catheter in place, were included in the study (Figure 1). The primary outcome was proven CRBSI. Procalcitonin measurements were obtained daily. CRBSI was suspected if patients met simultaneously all the following criteria: 1) clinical manifestations of infection (e.g., fever, chills, and/or hypotension) 2) central venous catheter being in place for more than 48 h and 3) no other apparent source of infection [6,23]. For suspected CRBSI, paired blood samples (10 ml) from catheter and a peripheral vein were obtained and together with a 5-cm segment of the catheter tip removed, were submitted for culture [6]. Subsequently, antibiotic therapy was initiated empirically, according to our local antimicrobial susceptibility data and included: meropenem, gentamicin and vancomycin.

Definitive diagnosis of CRBSI, according to published guidelines [6] required: 1.either the isolation of the same organism from one peripheral blood culture and the culture of the catheter tip (criterion 1), or 2. positive culture obtained from both the catheter hub and blood from peripheral vein (criterion 2), meeting CRBSI criteria for differential time to positivity (DTP) [6]. The semiquantitative (roll-plate) method was used for the catheters tip cultures with a colony count of 15 CFU/ml or more suggesting significant growth.

Exclusion criteria were any active infection upon ICU admission and infection other than CRBSI during ICU hospitalization (56 patients, Figure 1). If a patient had more than one episodes of suspected CRBSI during the study period, only the first episode was included in the study. In this respect, from the 46 remaining patients, 20 subjects were proven not to suffer a CRBSI (not proven CRBSI) and 26 were positive for catheter-related blood stream infection (proven CRBSI).

Acute Physiology and Chronic Health Evaluation (APACHE) II score and Sequential Organ Failure Assessment (SOFA) score of severity of illness were calculated upon admission and on a daily basis, respectively. Patients who had undergone any surgery in the 4 weeks preceding admission were considered surgical admissions. All other admissions were considered as medical. Patients were evaluated daily for clinical suspicion of infection and appropriate cultures from any potential site, as clinically indicated, were obtained, according to the principles of source control in the sepsis management [11]. SIRS and sepsis were defined according to published guidelines [24].

For management monitoring purposes patients with proven CRBSI were categorized in two subgroups depending on the infection control: a. Controlled, i.e. patients with controlled infection (n = 16), defined as clinical and microbiological improvement and b. Not controlled, i.e. defined as persistent bacteremia (the same pathogen responsible for episode 1 was cultured 72 h after catheter removal), superinfection (CRBSI due to another pathogen during therapy period), or death related to CRBSI (Figure 1). The empirical antibiotic therapy was considered appropriate if the isolated pathogen(s) was (were) susceptible to at least one drug administered at the onset of sepsis, according to the corresponding susceptibility test.

Day 0 (D0) was reported the first day of suspected CRBSI. The previous days were marked as (−). Duration of treatment of CRBSI was made following the relevant guidelines [6].

Blood samples for routine hematology and biochemistry panels, as well as PCT and CRP levels were measured within the first 24 h of admission, and then daily between 8.00 and 9.00 a.m. PCT was measured by the automated immuno-luminometric method (Liaison Brahms PCT, DiaSorin, Italy) with a detection limit of 0.03 ng/ml and a functional sensitivity of 0.24 ng/ml, according the manufacturer’s instructions. During treatment physicians in charge were not blinded but did not use PCT for clinical decision making.

### Statistical analysis

Statistical analysis of the data was performed using the SPSS, version 19.0 (SPSS, Inc., Chicago, IL, USA). The normality of quantitative variables was tested by Kolmogorov-Smirnov test. Normally distributed variables were expressed as mean ± standard deviation (SD), while non-normally distributed variables were expressed as median with interquartile range (IQR). The differences of all variables between groups were assessed by Student’s *t* test or Mann–Whitney *U* test, while within groups differences of PCT values were examined by Friedman test; post hoc analysis was performed using Bonferonni’s correction. Categorical variables were expressed as frequencies and percentages (%) and they were analyzed using the Chi-square test. The area under the receiver-operating-characteristics curve (ROC) was calculated to evaluate the diagnostic and prognostic significance of the tested parameters. Sensitivity, specificity, positive and negative predictive values were also calculated. The value with the shortest distance from the curve to the point with both maximum sensitivity and specificity, i.e., the point (0.0, 1.0), was selected as the optimal cut-off point, whereas AUC differences between studied parameters were assessed with Hanley and McNeil test. All tests were two tailed and a p < 0.05 was considered statistically significant for all analyses.

## Results

### Characteristics of the study population on admission

The demographic and clinical characteristics of patients on admission and final outcome are summarized in Table 1. No statistically significant differences were found in terms of age, gender, origin, causes of admission, APACHE II score, SOFA score, presence of SIRS, comorbidities, and ICU mortality. Furthermore, it was found that the two groups were comparable in terms of WBC count, CRP, and PCT values. In patients with proven CRBSI the ICU length of stay was significantly longer in comparison with patients with not proven CRBSI.

**Table 1:** Patient’s demographics and clinical characteristics on admission and final outcome

	**Not proven CRBSI**	**Proven CRBSI**	**P**
	**n = 20**	** n = 26**	
Age [years; mean (SD)]	44.45 (19.75)	51.73 (19.95)	0.224^a^
Male gender [no (%)]	10 (50.0)	18 (69.2)	0.185^b^
Origin [no (%)]			
Medical	9 (45.0)	12 (46.2)	0.938^b^
Surgical	11 (55.0)	14 (53.8)	
Reason of admission [no (%)]			
Neurological	13 (65.0)	12 (46.2)	0.203^b^
Respiratory	1 (5.0)	2 (7.7)	0.714^b^
Surgical	1 (5.0)	4 (15.4)	0.262^b^
Trauma	4 (20.0)	6 (23.0)	0.802^b^
Other	1 (5.0)	2 (7.7)	0.714^b^
APACHE II score [mean (SD)]	22.45 (4.32)	20.65 (4.69)	0.190^a^
SOFA score [mean (SD)]	7.15 (1.57)	6.81 (2.53)	0.598^a^
Presence of SIRS [no (%)]	6 (30.0)	9 (34.6)	0.741^b^
Co-morbidities			
Malignancy [no (%)]	1 (5.0)	2 (7.7)	0.714^b^
Diabetes mellitus [no (%)]	2 (10.0)	3 (11.5)	0.868 ^b^
Steroids [no (%)]	2 (10.0)	3 (11.5)	0.868^b^
Heart failure [no (%)]	1 (5.0)	2 (7.7)	0.714^b^
WBC count [10 ^3^/μl; mean (SD)]	14.47 (4.2)	12.80 (4.1)	0.185^a^
CRP [mg/dl; median (IQR)]	0.86 (0.50-2.65)	2.68 (0.62-10.88)	0.069^c^
PCT [ng/mL; median (IQR)]	0.10 (0.10-0.20)	0.10 (0.10-0.43)	0.168^c^
ICU length of stay [days; mean (SD)]	19.85 (6.99)	29.15 (11.34)	0.001^a^
ICU mortality [no (%)]	1 (5.0)	5 (19.2)	0.155^b^

^a^ Student’s *t* test.

^b^ Chi-square test.

^c^ Mann–Whitney test.

### Microbiology results

Among the 26 patients with proven CRBSI, 22 (84.6%) exhibited gram–negative bacteremia and 6 (23%) gram–positive bacteremia. Microorganisms considered responsible for CRBSI were: Acinetobacter baumanii (n = 12), Klebsiella pneumoniae (n = 6), Pseudomonas aeruginosa (n = 3), E coli (n = 1), methicillin-resistant Staphylococcus aureus (n = 1), methicillin-sensitive Staphylococcus aureus (n = 1), and coagulase-negative staphylococci (n = 4). Two patients had more than one pathogen.

Group with patients suffering not controlled infection included: 3 patients with persistent bacteremia, 2 patients with persistent bacteremia and superinfection, and 5 patients who died because of CRBSI. The isolated pathogens of patients with not controlled infection were mainly Gram-negative organisms and included: Klebsiella pneumoniae (3 cases), Pseudomonas aeruginosa (3 cases), Acinetobacter baumanii (3 cases); only one patient exhibited Gram-positive bacteremia (Coagulase-negative staphylococcus). One-half of patients with not controlled infection due to Gram-negative bacteria were given inappropriate empirical antibiotics within the first 3 days of sepsis management, whereas empirical antibiotic therapy was appropriate in all patients with controlled CRBSI.

### Day of infection (D0)

Clinical characteristics of the 2 groups of patients on the day of infection suspicion (D0) are presented in Table 2. Time of CVC being in place and time elapsed from ICU admission until D0 did not differ between the 2 groups of patients. Moreover, patients did not differ in terms of presence of SIRS, development of shock, and administration of systemic antibiotics. Among studied biomarkers, only PCT serum levels were found significantly increased in patients with proven CRBSI compared with patients with not proven CRBSI [7.70 (2.50-11.43) vs. 0.10 (0.10-0.27), (p < 0.001)]. A tendency towards higher severity of illness was found in patients with proven infection (p = 0.087). Among patients with proven CRBSI 6 fulfilled the diagnostic criterion 1, 10 the criterion 2, and the remaining 10 patients both the two criteria (Table 2).

**Table 2:** Patient’s clinical characteristics on the day of infection (D0), PCT kinetics the previous 4 days until D0 and diagnostic criteria for CRBSI

	**Not proven CRBSI**	**Proven CRBSI**	**P**
	**n = 20**	** n = 26**	
Time elapsed from ICU admission [days; mean (SD)]	10.70 (2.62)	11.77 (3.31)	0.242^a^
Catheter in place [days; mean (SD)]	8.25 (1.41)	8.73 (2.05)	0.375^a^
Presence of SIRS [no (%)]	20 (100)	26 (100)	1.0 ^b^
WBC count 10 ^3^/μl; mean (SD)]	14.68 (3.8)	15.37 (6.03)	0.658^a^
CRP [mg/dl; median (IQR)]	12.75 (7.93-18.33)	15.78 (10.59-21.48)	0.236^c^
SOFA score [mean (SD)]	5.85 (2.41)	7.19 (2.70)	0.087^a^
Shock [no (%)]	9 (45.0)	14 (53.8)	0.552^b^
PCT [ng/ml; median (IQR)]			
D-4 (20/26) ^d^	0.15 (0.10-0.20)	0.20 (0.10-0.50)	0.289^c^
D-3 (20/26) ^d^	0.10 (0.10-0.28)	0.25 (0.10-0.63)	0.017^c^
D-2 (20/26) ^d^	0.10 (0.10-0.20)	0.50 (0.20-1.40)	<0.001^c^
D-1 (20/26) ^d^	0.10 (0.10-0.30)	0.75 (0.40-2.50)	<0.001^c^
D0 (20/26) ^d^	0.10 (0.10-0.27)	7.70 (2.50-11.43)	<0.001^c^
ΔPCT_D-4, D0_	0.00 (−0.90-0.40)	7.05 (2.40-10.43)	<0.001^c^
ΔPCT_D-3, D0_	0.00 (−0.90-0.40)	6.45 (2.40-10.30)	<0.001^c^
ΔPCT_D-2, D0_	0.00 (−0.60-0.40)	5.55 (2.23-9.93)	<0.001^c^
ΔPCT_D-1, D0_	0.00 (−0.40-0.10)	4.85 (1.00-9.28)	<0.001^c^
Diagnosis of CRBSI			
Criterion 1 [no (%)]	_	6 (23.1)	
Criterion 2 [no (%)]	_	10 (38.5)	
Both criteria (1 + 2) [no (%)]	_	10 (38.5)	

^a^ Student’s *t* test; ^b^ Chi-square test; ^c^ Mann–Whitney test; ^d^ No of patients available for analysis (Not proven/proven).

### Days D-4 to D0

As shown in Table 2, PCT serum levels were compared between the two groups of patients in the 4 days preceding D0. Although, no significant differences were found during D-4 (p = 0.289), PCT was significantly higher between patients with proven CRBSI during D-3 (p = 0.017), D-2 (p < 0.001), and D-1 (p < 0.001), in relation with patients with not proven CRBSI. In addition, longitudinal changes of PCT concentration between D0 and the four preceding days were also significantly higher in patients with proven CRBSI than in those with not proven CRBSI (ΔPCT_D-4, D0_: p < 0.001; ΔPCT_D-3, D0_: p < 0.001; ΔPCT_D-2, D0_: p < 0.001; ΔPCT_D-1, D0_: p < 0.001).

**Figure 2:**
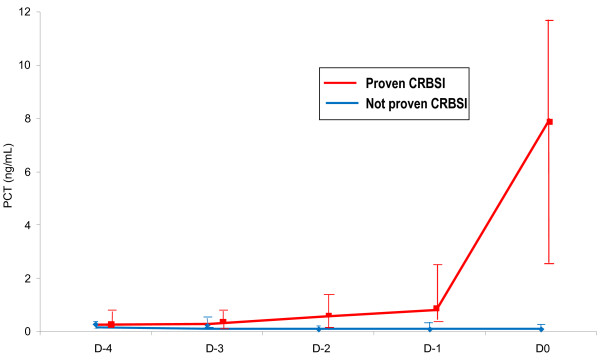
**PCT kinetics of patients with and without proven CRBSI from Day-4 (D-4) to Day 0 (D0).** Results are expressed as median values with IQR (25-75%).

PCT time course between D0 and the 4 preceding days for the two groups of patients are shown in Figure 2. Friedman test revealed that variation over time of PCT levels was statistically significant within patients with proven infection (p < 0.001), but not within patients with not proven CRBSI (p = 0.415). In particular, for the group with proven CRBSI, post hoc analysis showed that the 25% elevation of marginal significance (p = 0.078) of PCT from D-4 to D-3, was followed by a stepwise statistically significant increase from D-3 to D0 (100% from D-3 to D-2, p = 0.006; 50% from D-2 to D-1, p < 0.001; 927% from D-1 to D0, p < 0.001).

### Diagnostic significance - ROC analysis

**Figure 3:**
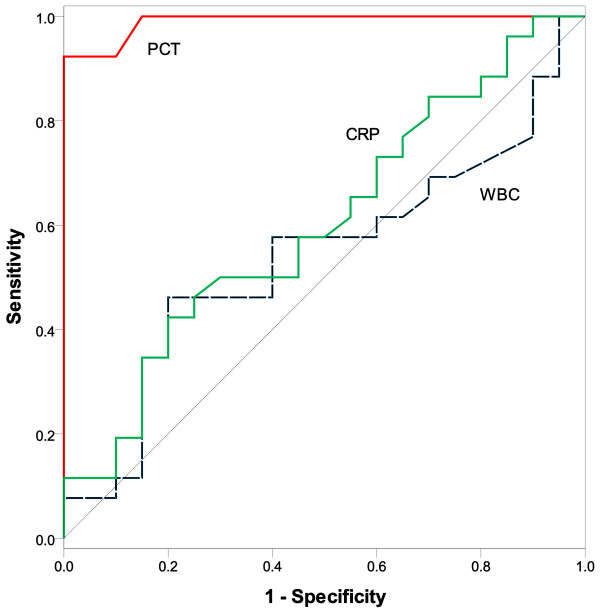
ROC curves of PCT, WBC, and CRP on D0 for differentiating between patients with and without proven CRBSI in the ICU.

Figure 3 and Table 3 show the diagnostic accuracy of all studied biomarkers for proven CRBSI on D0, estimated with the area under the curve (AUC). The AUC for PCT was 0.990 (95% CI, 0.972 – 1.000), for WBC 0.539 (95% CI, 0.369 – 0.709), and for CRP 0.603 (95% CI, 0.438 – 0.768). Furthermore, it was found that the AUC for PCT was significantly higher compared to the AUCs for WBC (p < 0.001) and CRP (p < 0.001) serum levels. The optimal cut-off points for these diagnostic markers were also determined by the ROC curve (Table 3). In particular, an optimal cut-off point of 0.70 ng/ml for PCT on D0 was found to discriminate patients with and without CRBSI, with a sensitivity of 92.3% and a specificity of 100%. A 100% Sensitivity was obtained for a cut-off of 0.40 ng/ml (with specificity of 85%). The cut-offs for WBC and CRP serum levels were 15.64 10^3^/μl and 16.90 mg/dl respectively and yielded moderate sensitivities (WBC: 57.7%; CRP: 50%) and specificities (WBC: 60%; CRP: 70%). PCT variations were also found to be predictive of proven CRBSI. Thus, an increase >0.20 ng/ml of PCT between the D0 and any of the 4 preceding days was associated with sensitivities 96 to 100%, and specificities (all) 95%. Interestingly, 4 patients without proven CRBSI who underwent various minimal surgical procedures (2 cases with transcutaneous tracheotomy and 2 cases with intracranial pressure placement) had a rise in their PCT (≤ 0.20 ng/ml) between D0 and the 4 preceding days.

**Table 3:** Diagnostic significance of PCT, PCT kinetics, WBC, and CRP for CRBSI during ICU stay

	**AUC (95% CI)**	**Cut-off**	**Sensitivity (%)**	**Specificity (%)**	**PPV (%)**	**NPV (%)**	**LR(+)**	**LR(−)**	**Accuracy (%**)
PCT D0 [ng/ml]	0.990 (0.972 – 1.000)	>0.70	92.3 (73.4 – 98.7)	100 (80.0 – 100)	100.0	90.9	n.a.	0.08	95.7
ΔPCT_D-1, D0_ [ng/ml]	0.982 (0.941 – 1.000)	>0.20	100 (84.0 – 100)	95.0 (73.1 – 99.7)	96.3	100.0	20.0	0.0	97.8
ΔPCT_D-2, D0_ [ng/ml]	0.992 (0.975 – 1.000)	>0.20	100 (84.0 – 100)	95.0 (73.1 – 99.7)	96.3	100.0	20.0	0.0	97.8
ΔPCT_D-3, D0_ [ng/ml]	0.980 (0.939 – 1.000)	>0.20	96.2 (78.4 – 99.8)	95.0 (73.1 – 99.8)	96.2	95.0	19.2	0.04	95.6
ΔPCT_D-4, D0_ [ng/ml]	0.982 (0.946 – 1.000)	>0.20	96.2 (78.4 – 99.8)	95.0 (73.1 – 99.8)	96.2	95.0	19.2	0.04	95.6
WBC D0 [10 ^3^/μl]	0.539 (0.369 – 0.709)	>15.64	57.7 (37.2 – 76.0)	60.0 (36.4 – 80.0)	65.2	52.2	1.44	0.71	58.7
CRP D0 [mg/dl]	0.603 (0.438 – 0.768)	>16.90	50.0 (30.4 – 69.6)	70.0 (45.7 – 87.2)	68.4	51.9	1.67	0.71	58.7

AUC (95% confidence interval, CI), positive (PPV) and negative (NPV) predictive value, LR(+) likelihood ratio positive, and LR(−) likelihood ratio negative.

### Prognostic value of PCT

**Figure 4:**
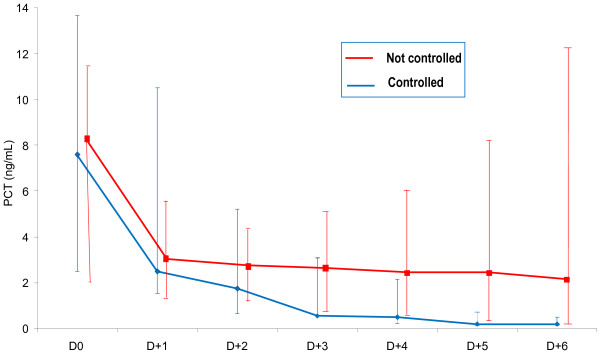
**PCT kinetics of controlled and not controlled patients with CRBSI from Day 0 (D0) to Day 6 (D + 6).** Results are expressed as median values with IQR (25-75%).

Patients with and without controlled CRBSI were compared in terms of PCT concentrations during the 6 following days after D0 (Figure 4). A highly significant reduction of PCT concentration on D(1) was observed in both groups (p = 0.002 in controlled, p = 0.005 in not controlled). Subsequently, PCT serum levels tended to further decrease significantly in patients with controlled CRBSI, whereas in not controlled patients was proven to remain stable. D3 was the first day during therapy, where a statistically significant difference was found between the two subgroups of patients (Table 4). Specifically, on D3 a concentration of PCT more than 1.5 ng/ml was associated with non-response to therapy with a sensitivity 70% and specificity 68.7% (p = 0.028). PCT variations were also found to be predictive of non-response to therapy (Table 4). Thus, a decrease of PCT concentration between D1 and D2 (ΔPCT D1- D2) of more than 1.00 ng/ml, and between D2 and D3 (ΔPCT D2-D3) exceeding 0.30 ng/ml were associated with good response to therapy.

**Table 4:** Patient’s clinical characteristics on the day of infection (D0) and PCT kinetics the next 6 days after diagnosis of CRBSI

	**Controlled**	**Not controlled**	**P**
	**n = 16**	**n = 10**	
WBC count [10^3^/μl; mean (SD)]	15.69 (6.9)	14.87 (4.5)	0.745^a^
CRP [mg/dl; median (IQR)]	15.78 (10.06-21.05)	15.65 (10.55-25.75)	0.732^c^
SOFA score [mean (SD)]	6.56 (2.19)	8.20 (3.22)	0.135^a^
Shock [no (%)]	7 (43.8)	7 (70.0)	0.191^b^
PCT [ng/mL; median (IQR)]			
D0 (16/10) ^d^	7.55 (2.50-13.67)	8.30 (2.07-11.35)	0.771^c^
D + 1 (16/10) ^d^	2.50 (1.53-10.52)	3.00 (1.30-5.55)	0.635^c^
D + 2 (16/10) ^d^	1.75 (0.65-5.22)	2.70 (1.22-4.27)	0.635^c^
D + 3 (16/10) ^d^	0.55 (0.50-3.10)	2.60 (0.75-5.12)	0.047^c^
D + 4 (16/10) ^d^	0.50 (0.23-2.15)	2.40 (0.67-6.00)	0.042^c^
D + 5 (16/10) ^d^	0.20 (0.13-0.72)	2.40 (0.35-8.17)	0.009^c^
D + 6 (16/9) ^d^	0.20 (0.12-0.50)	2.10 (0.20-12.25)	0.012^c^
ΔPCT_D0, D+1_	−2.65 (−8.90 to −0.75)	−4.60 (−5.63 to −0.78)	0.895^c^
ΔPCT_D+1, D+2_	−1.65 (−5.45 to −0.38)	−0.30 (−1.30 to −0.08)	0.037^c^
ΔPCT_D+2, D+3_	−1.00 (−2.68 to −0.15)	−0.05 (−0.45 to 0.25)	0.017^c^
ΔPCT_D+3, D+4_	−0.15 (−1.65 to −0.03)	−0.15 (−0.28 to 0.73)	0.253^c^
ΔPCT_D+4, D+5_	−0.25 (−0.68 to −0.12)	−0.10 (−0.33 to 0.75)	0.085^c^
ΔPCT_D+5, D+6_	0.00 (−0.10 to 0.08)	0.00 (−0.45 to 0.20)	0.872^c^

^a^ Student’s *t* test; ^b^ Chi-square test; ^c^ Mann–Whitney test; ^d^ No of patients available for analysis (Controlled/Not controlled).

## Discussion

The principal findings of this study performed in selected critically ill patients with suspected CRBSI are: (1) the confirmation of good diagnostic accuracy of PCT; (2) the modest discriminative value of other indicators of inflammation, such as WBC, and CRP; and (3) the good prognostic performance of PCT and PCT kinetics as a monitoring tool to infection control during antimicrobial therapy.

Although there is accumulating evidence that PCT is not a “magic biomarker” [19], many studies have shown its ability to early detect bacterial infections in critically ill patients [18]. However, this is the first prospective observational study assessing accuracy of PCT for the diagnosis of CRBSI in the ICU.

According to our results, the level of PCT obtained during the day of suspected CRBSI is a better diagnostic marker compared with WBC and CRP. Additionally, on the day of clinical suspicion of infection a cut-off point of 0.7 ng/ml for PCT was proven to discriminate patients with and without CRBSI.

Similar results were found in other selected populations [21,22], where a cut-off of 0.1 ng/ml PCT was found to have 100% sensitivity in excluding contamination from bloodstream infection due to coagulase-negative Staphylococci [22].

Although the most commonly reported causative pathogens for CRBSI remain coagulase-negative staphylococci [6], in our cohort gram-negative bacteria were predominantly recovered from patients with proven CRBSI. This may be due to the fact that our patients were critically ill with prolonged ICU length of stay (Table 2). Since this special group of patients is usually colonized with gram-negative multi-resistant bacteria, the vast majority of CRBSI have been found to be of gram-negative origin [15,25]. It has been shown that in critically ill patients, GN bacteremia could be associated with higher PCT values than those found in GP bacteremia [26]. This could account for the fact that in our study, PCT elevations were higher than those from the study of Schuetz et al. [22].

It is well established that non-specific elevations of PCT serum levels in the absence of a bacterial infection can be seen in situations of severe stress, such as trauma or postoperatively [12]. Because more than half of our patients were of surgical origin, we considered that the assessment of PCT kinetics could overcome this drawback. In this respect, we have shown that an increase of PCT more than 0.20 ng/ml, between the day of clinical suspicion of CRBSI and any of the 4 preceding days, is associated with significant diagnostic accuracy for CRBSI (sensitivities > 96%, and specificities 95%).

Our findings are similar with those reported in two recent studies that tried to assess PCT’s diagnostic value for early detection of different infections in the ICU [14,15].

However, the authors did not evaluate specifically CRBSI, and a proportion of their patients had been suffering previous septic episodes.

In addition, the diagnostic accuracy of PCT in our study was better compared with findings from the previous studies [14,15]. We believe that this may be due to several factors. First of all, our patients were not suffering from any previous infection during enrollment in the study, a criterion that may enhance the diagnostic performance of PCT [27]. Moreover, only a small proportion of our patients’ population (23%) was receiving antibiotics at the time of suspected infection. This practice may affect the interpretation of PCT as a diagnostic method [27,28]. Furthermore, we have shown that patients with proven CRBSI had significantly higher PCT levels not only during D0, but also during the three preceding days. This increase of PCT prior to the clinical manifestation of infection is an important finding of our study and parallels results from Schuetz et al. [22]. Interestingly, as they concluded “this early increase may reflect the colonization of the catheter with subclinical infection eventually leading to CRBSI” [22].

Charles et al. found a significant decrease of PCT between the second and third day after the onset of sepsis in critically ill patients treated with appropriate antibiotic therapy [29]. In the present study, it has been demonstrated that PCT kinetics during the first 3 days of therapy could discriminate responders from non-responders, in terms of antimicrobial treatment. Moreover, a PCT concentration exceeding 1.5 ng/ml on the third day of treatment seems to be prognostic for lack of response to therapy.

Some limitations of our study should be noted. First, population sample was relatively small, so these results need to be confirmed in larger studies. Second, the included patients were relatively young, with a low rate of underlying disease. In addition they were mainly of surgical origin with neurological and trauma admission diagnosis. Consequently, the findings of our study cannot be extrapolated to other groups of critically ill patients. In addition, we had excluded all patients with any other infection during ICU hospitalization and used a control group without infection. In this respect, our study population included patients that were clearly different from those the ICU physician use to deal with in the ICU setting, where patients might have fever from different infectious and non- infectious causes. Although this limitation represents a weakness of our study, on the same time we believe is one of its strength, since withdrawn of potential confounders may increase usefulness of different biomarkers, as diagnostic and monitoring tools, in daily practice. Therefore, because of the lack of a comparative group having fever due to other etiologies, the true accuracy of PCT in ICU patients with CRBSI need to be confirmed in larger studies including more complex patients with fever and systemic inflammation. In addition, since the diagnostic accuracy of PCT and its optimal cut-offs are dependent on the use of a sensitive assay [12], the fact that the Kryptor test was not available for serum PCT measurement, is another limitation of our study. Finally, the high PCT levels observed in patients with CRBSI could be the result of another concurrent infection. However, this seems unlikely since every effort was made to early identify and exclude patients with other causes of infection, even after proven CRBSI diagnosis.

In conclusion, we suggest that PCT could be an appropriate method for diagnosis of CRBSI. In addition, its adoption in every day clinical practice could prevent unnecessary withdrawing of thousands of central venous catheters, limiting at the same time inappropriate use of empirical antimicrobial therapy. Moreover, PCT seems to have some prognostic value regarding therapeutic monitoring. Thus, its course, even in the first 3 days of therapy, could predict non-responders with CRBSI, and might help clinicians to make a proper and early decision concerning therapeutic alternatives (i.e., resistance to administered empirical antibiotic treatment needing shift to another therapeutic combination or possibly complicated CRBSI, such as development of suppurative thrombophlebitis, urging a more extended diagnostic work-up).

## Conclusions

We suggest that PCT serum levels and PCT kinetics could serve as early and accurate markers for the diagnosis and prognosis of CRBSIs in the ICU setting, associated with clinical findings. However, larger observational studies, and clinical trials would be the best way to demonstrate the impact of PCT in ICU patients with suspected CRBSI. Moreover, our results need external validation in a more heterogeneous group of patients with other possible etiologies of infection and systemic inflammation.

## Abbreviations

PCT: Procalcitonin; CRBSI: Catheter-related bloodstream infections; ICU: Intensive care unit; AUC: Area under curve; WBC: White blood cells; CRP: C-reactive protein; CVC: Central venous catheters; SIRS: Systemic inflammatory response syndrome; APACHE: Acute Physiology and Chronic Health Evaluation; SOFA: Sequential Organ Failure Assessment; DTP: Differential time to positivity; SD: Standard deviation; IQR: Interquartile range; CI: Confidence interval; LR: Likelihood ratio; ROC: Receiver-operating-characteristics curve; VAP: Ventilator-associated pneumonia.

## Competing interests

The authors declare that they have no competing interests.

## Authors’ contributions

VPT was the principal investigator who designed the study, collected data, and wrote the manuscript. VEP reviewed, edited the manuscript, and participated in the coordination of the study. GAT performed the statistical analysis of the data, and participated to their interpretation. MKP and TMG managed the laboratory measurements. EKC and GAK collected the data, and helped to draft the manuscript. ESM, SIKK, and IAP supervised the whole study. All authors read and approved the final manuscript.

## Pre-publication history

The pre-publication history for this paper can be accessed here:

http://www.biomedcentral.com/1471-2334/12/247/prepub
